# Anatomical studies on the PES region of Zebu cattle (*Bos Taurus indicus*) with special references to 3D computed tomography imaging technique

**DOI:** 10.1186/s12917-024-03940-0

**Published:** 2024-03-08

**Authors:** Ahmed Nomir, Ashraf El Sharaby, Mohamed M. A. Abumandour

**Affiliations:** 1 Department of Anatomy and Embryology, Faculty of Veterinary Medicine, Damanhour University, Damanhour, Egypt; 2 Department of Anatomy and Embryology, Faculty of Veterinary Medicine, Alexandria University, Post Box: 22758, Alexandria, Egypt

**Keywords:** Anatomy, Computed tomography, 3D render volume reconstruction CT (*3D-RVCT*), Pes, Zebu bulls

## Abstract

The 3D render volume reconstruction CT (3D-RVCT) produced detailed images of the PES region, determining its relationships with the surrounding structures. Despite extensive research in veterinary studies on the PES through gross anatomy and CT, there is a lack of studies on the PES of zebu cattle. The study aimed to analyze the PES of Zebu cattle using gross cross-sectional, radiographic, CT, and morphometric methods, with the use of 3D-RVCT to provide anatomical guidance for surgeons and students. The study was performed on sixteen PES regions to provide hard and soft tissues in CT images. Three are five tarsal bones and two large fused (III and IV) metatarsal bones that were completely fused except for their distal extremities, which were divided distally by the intertrochlear notch. The cortical thickness of the metatarsal bone was equal on both sides. The bony septum divided the medullary cavity between the two fused large metatarsal bones in the proximal distal half only and disappeared in the middle part. The reconstruction showed similar sizes in the right and left limbs, confirming the pes bones. The radiographic and CT images could be used as a normal reference for the interpretation of some clinical diseases in the PES. The 3D CT reconstruction of the pes bones was described by various CT oblique dorsal and plantar views. The study focuses on diagnosing PES disorders using CT imaging, improving medical interventions, improving Zebu cattle health outcomes, and empowering students to contribute to veterinary medicine research and advancements.

## Highlight

The study aimed to analyze the PES region of Zebu cattle using various methods, including 3D-RVCT, to provide anatomical guidance for surgeons and students. The study focused on sixteen PES regions, including three tarsal bones and two large fused metatarsal bones. The cortical thickness of the metatarsal bone was equal on both sides, and the bony septum divided the medullary cavity between the two fused large metatarsal bones in the proximal distal half only. The reconstruction showed similar sizes in the right and left limbs, confirming the pes bones. The radiographic and CT images could be used as a normal reference for interpreting clinical diseases in the PES. The 3D CT reconstruction of the pes bones was described by various CT oblique dorsal and plantar views. The study aims to diagnose PES disorders using CT imaging, improve medical interventions, improve Zebu cattle health outcomes, and empower students to contribute to veterinary medicine research and advancements.

## Introduction

Zebu or indicine cattle* (Bos Taurus indicus)*, first described by Linnaeus in 1758, is a subspecies of the *Bos Taurus Bos* Genus, family *Bovidae*, and is also referred to as the humped cattle. The Zebu were distinguished by a fatty hump in front of their shoulder region, which they developed as they adapted for tropical environments and high temperatures [1, 2]. Zebu cattle produce very little milk and are bred primarily for meat [2].

The locomotor system is the most significant system in all animal species, which gives more anatomical attention to studying its anatomical features, including the joints. The ruminant’s skeleton system is essential to the anatomical description due to their high muscle mass. With a few minor comparisons to ruminants, the appendicular skeleton of horses is described in basic normal gross osteology in anatomical textbooks [3–5]. Only a few anatomical textbooks [3–6] and recently published research [7–10] have described the typical gross anatomy of the appendicular skeleton in ruminants. The metatarsal bone, which is crucial to the ruminant skeletal system, is formed by the full union of the metatarsal bones III and IV [10].

The availability of computed tomography (CT) equipment at academic institutions and private veterinary hospitals has led to an increase in the use of computed tomography (CT) techniques in veterinary medicine [11]. The new anatomical techniques, such as radiology, computed tomography, MRI, and other techniques, are useful for students, anatomists, and surgeons. Imaging anatomical techniques had a significant function in the description of the animals' feet and plays a great role in contemporary biological research [8, 9, 12, 13]**.** CT is a particularly helpful method for displaying generic anatomy due to its high spatial resolution and modest differentiation of tissue contrast [14].

When examining complicated bone structures like the spine, joints, or skull, CT is especially helpful. Incorporating modern methods into anatomy guides in Egypt is crucial for adhering to international standards and preparing students for the clinical phase of veterinary study [9, 12, 13, 15]. These methods include digital X-ray images, computed tomography (CT), magnetic resonance imaging (MRI), ultrasonography, endoscopy, fluoroscopy, 3D images, animation, and CDs. It is possible to diagnose abnormalities of the foot and foot pad more accurately with the use of CT, an effective imaging technique that offers a cross-sectional image with superior soft tissue distinction and no superimposition of the overlaying structures [12, 13, 16]. Due to its affordability, accessibility, and comprehensive examination of bony structures, radiography continues to be the primary modality of equine musculoskeletal imaging [12, 13, 17].

In the veterinary published articles, there are a number of publications describe the normal CT anatomy of numerous parts of the body to give more benefit anatomical details on the structure as; limbs [16, 18, 19], heads [15, 20–27], thorax [28–30], abdomen [31, 32], and pelvic [33]. Clinical anatomy is one of the main principles of clinical and surgical practice because it enables the clinician to visualize details of structures relevant to the case at hand [5].

There is a paucity of anatomical information about the bones, muscles, ligaments, and joints of the pes region of Zebu bulls (*Bos Taurus indicus*). The current study's goal was to provide a complete gross, radiographic, cross-sectional, computed tomography (CT), and morphometric analysis of the musculoskeletal components (bones, muscles, ligaments, and joints) of the pes region of Zebu bulls (*Bos Taurus indicus*). The obtained data were then compared to information on various animal species that had previously been published.

## Material and methods

### Sample collection

Our study was carried out on sixteen cadaveric distal limbs (pes regions) of adult healthy and clinically normal Zebu beef fattening bulls (*Bos Taurus indicus, Linnaeus, 1758*) aged 12–18 months and weighing 400–500 kg, in which the age of the Zebu bulls was determined by dentation according to TF Best [34]. These limbs were obtained from a semiautomatic slaughterhouse of the Egyptian army, Fayoum governorate (Egypt) by the veterinarian in a local slaughterhouse. The animals were slaughtered for meat consumption, not for experimental research purposes. The pes regions were collected at the slaughterhouse, placed on ice, and immediately transferred to the laboratory. All the specimens were cleansed with tap water, cooled, and prepared for X-ray and CT imaging. The collected samples must be free from any musculoskeletal disorders. The Zebu beef bulls were examined clinically by palpations of the carpal, metacarpal, and pes regions for lameness before their slaughtering. The pes regions were separated from the hind limbs just at the tarsal joint, kept frozen at -20 °C, left solidified until being processed for sectional anatomy at different planes, and imaged within two hours to avoid postmortem changes. The collected pes regions were kept in a normal saline solution in ambient air for two hours.

This study was carried out with ethical permission from the faculty of Veterinary Medicine, Alexandria University, and approved by the Institutional Animal Care and Use Committee (ALEXU-IACUC) and the faculty of Veterinary Medicine, Damanhour University, and approved by Institutional Animal Care and Use Committee (IACUC) with (Approval code: DMU/VetMed-2023/038). All methods were performed in accordance with relevant guidelines and regulations from the Basel Declaration and the International Council for Laboratory Animal Science (ICLAS). The anatomical nomenclature was applied according to *Nomina Anatomica Veterinaria* [35].

### Imaging techniques

Six specimens were subjected to X-ray and CT scanning within two hours after slaughtering to minimize post-mortem changes [36].

#### Radiography (X-ray imaging)

Prior to CT examination, the collected specimens were radiographed for the normal morphology of the musculoskeletal components. Each pes was properly positioned in its planter aspect on the scanning table to collect dorsoplantar radiographic views. Then, it was positioned on the lateral aspect for screening mediolateral views. Radiographic views were made by the Toshiba 500 mA and FCR Prima T2 X-ray devices (Toshiba, Minato, Tokyo, Japan) with an output of 80 kV and 320 mAs in the dorsal, lateral, and planter aspects, and hard copies for the dorsoplantar and mediolateral radiographic views were printed out on x-ray films. Soft copies were also obtained for offline surveying of the PES region.

#### Computed tomography (CT)

**Fig. 1:**
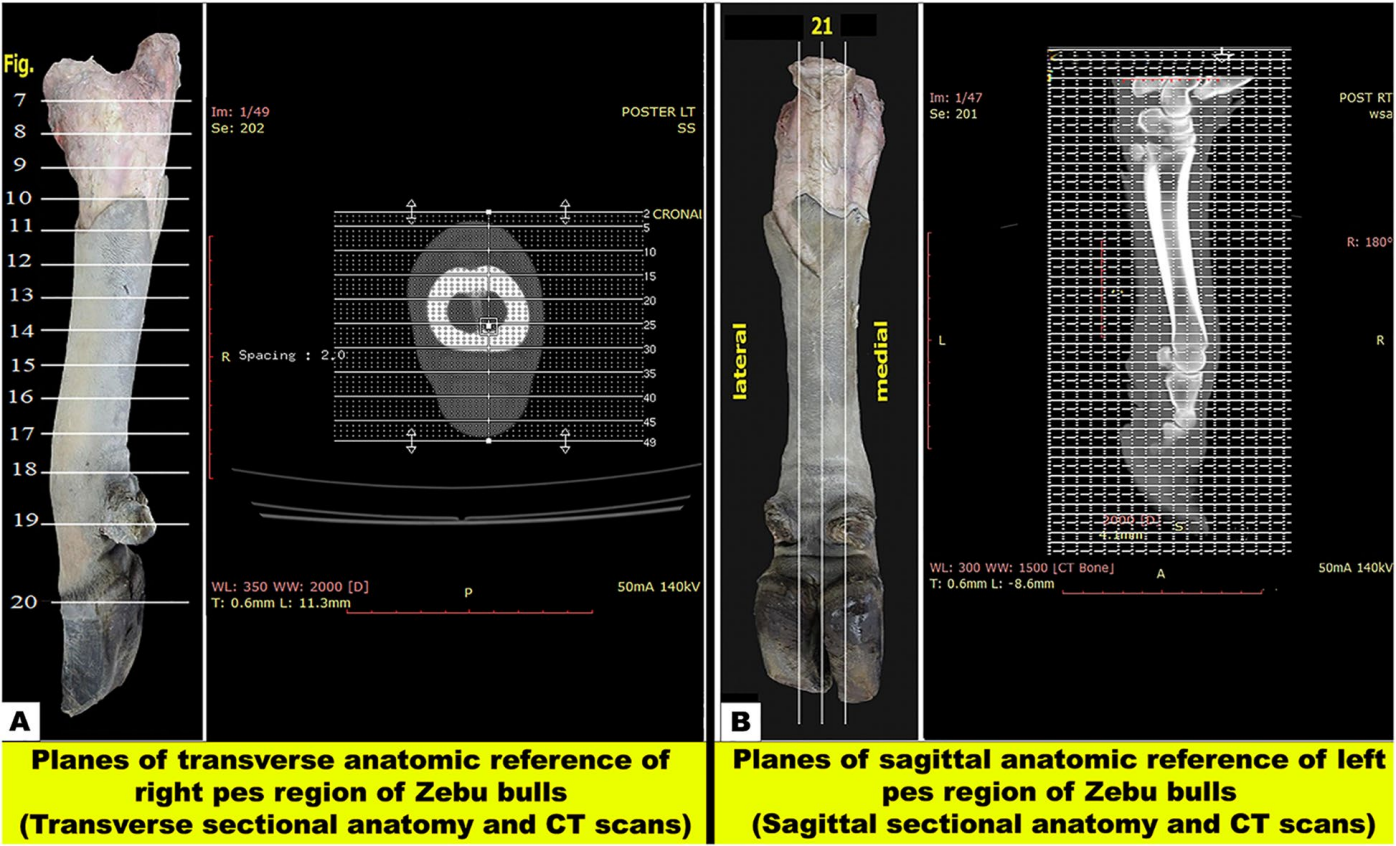
Anatomical planes of the pes region of Zebu bulls. View **A** shows the planes of the transverse anatomic reference of the right pes region (transverse sectional anatomy and CT scans). Fourteen serial transverse sections were cut starting from the level of the tibiotarsus joint (Fig. 3A and B) and continuing distally until the level of the middle of the third phalanx (Fig. 9). View **B** shows the planes of the sagittal anatomic reference of the left pes region of Zebu bulls (sagittal sectional anatomy and CT scans). One sagittal section was cut at about the midline of the specimen (Fig. 10A–C)

**Fig. 2:**
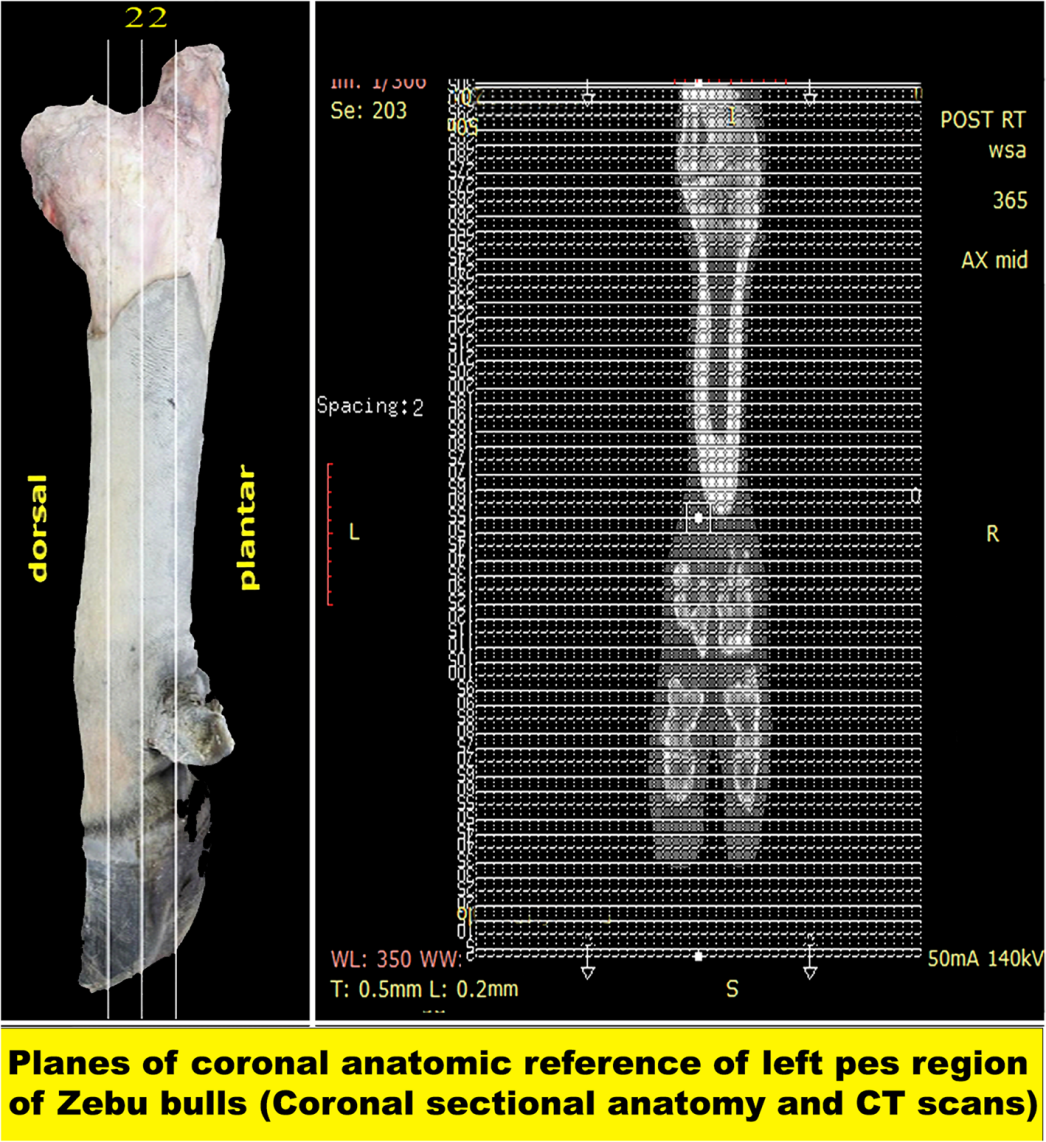
Planes of coronal anatomic reference of the left pes region of Zebu bulls (coronal CT sectional anatomical scans). One coronal section was obtained from a third limb at about the middle (Fig. 10D–F)

The collected specimens were positioned in their plantar aspect on the scanning table, and tomograms were made perpendicular to the proximodistal longitudinal axis to get transverse slices (Fig. 1A), the mediolateral axis for sagittal slices (Fig. 1B), and finally changed alongside the dorsoplantar axis to get coronal slices (Fig. 2). Contiguous CT images were obtained in helical scan mode using a 128-detetcor, multi-slice (16-slice), and spiral Optima CT520 Series scanner (Siemens Healthiness CT device (Aquilion; Toshiba Medical Systems, Tokyo, Japan); scanning conditions: 140 kV, 50 mA, 4 s, window width; WW: 400; window level WL 60 Hounsfield units) set for soft window. A bone window was obtained after changing settings (window width, WW: 1,500; window level, WL: 300 Hounsfield units). Three-dimensional CT imaging of the pes skeleton was performed using the same CT system after changing its settings (window width, WW: 332 window level, WL: 287 Hounsfield units). For documentation, the scanned CT images were saved and then printed using a CT digital printer, and the digital workstation images were saved on a hard drive for offline investigation using Radiant DICOM Viewer software (version 2020.2.3) (Medixant, Poznan, Poland) to reconstructed these images in multiplanar reconstructed (MPR) and three dimensions (3D).

**Fig. 3:**
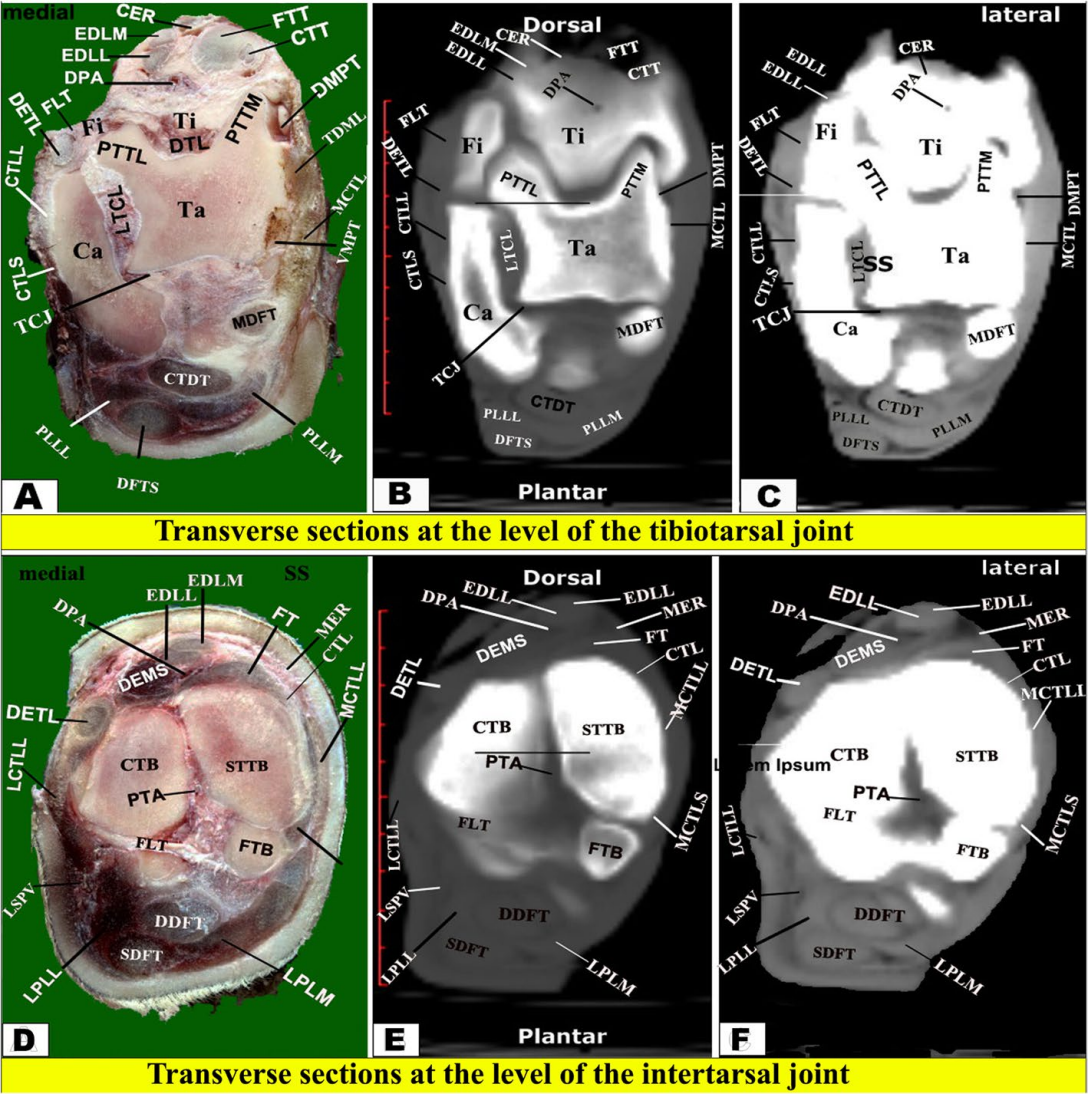
Transverse sectional-anatomy of the pes region of Zebu bulls. Views **A** and **D** Gross and Views **B**–**C** and **E**–**F** CT sections. Views **A**–**C** represent the transverse sections at the level of the tibiotarsal joint (Gross, bone windows, and soft windows). Views **D**–**F** represent the transverse sections at the level of the intertarsal joint (Gross, bone windows, and soft windows). to show the (Ca) Calcaneus; (CER) Crural extensor retinaculum; (CTLS) Collateral tarsal ligament (short part); (CTB) Central tarsal bone; (CTDT) Common tendon of the lateral digital flexor; (CTT) Cranial tibial tendon; (DMPT) Dorsomedial pouch of the tarsocrural joint; (DPA) Dorsal pedal artery; (DTL) Dorsal tarsal ligament; (DDFT) Deep digital flexor tendon; (DETL) Digital extensor tendon (lateral part); (EDLL) Extensor digitorum longus (lateral part); (EDLM) Extensor digitorum longus (medial part); (DMPT) Dorsomedial pouch of the tarsocrural joint; (FTB) First tarsal bone; (STTB) Second and third tarsal bone; (FT) fibularis tertius; (Fi) Fibula; (FLT) fibularis longus tendon; (FTT) Fibularis tertius tendon; (LCTLL) lateral collateral tarsal ligament (Long part); (LCTLL) lateral collateral tarsal ligament (Long part); (LSPV) Lateral saphenous vein and lateral planter vein (caudal branch); (LPLL) Long planter ligament (lateral part); (LPLM) Long planter ligament (medial part); (MCTL) Medial collateral tarsal ligament (long part); (MDFT) Medial digital flexor tendon; (PTTL) Proximal trochlea of talus (lateral part); (MER) Metatarsal extensor retinaculum; (PTTM) Proximal trochlea of talus (medial part); (Ta) Talus; (TCJ) Talocalcaneal joint; (TDML) Talocentro-distometatarsal ligament; (Ti) Tibia; and (VMPT) Ventromedial pouch of tarsocrural joint

**Fig. 4:**
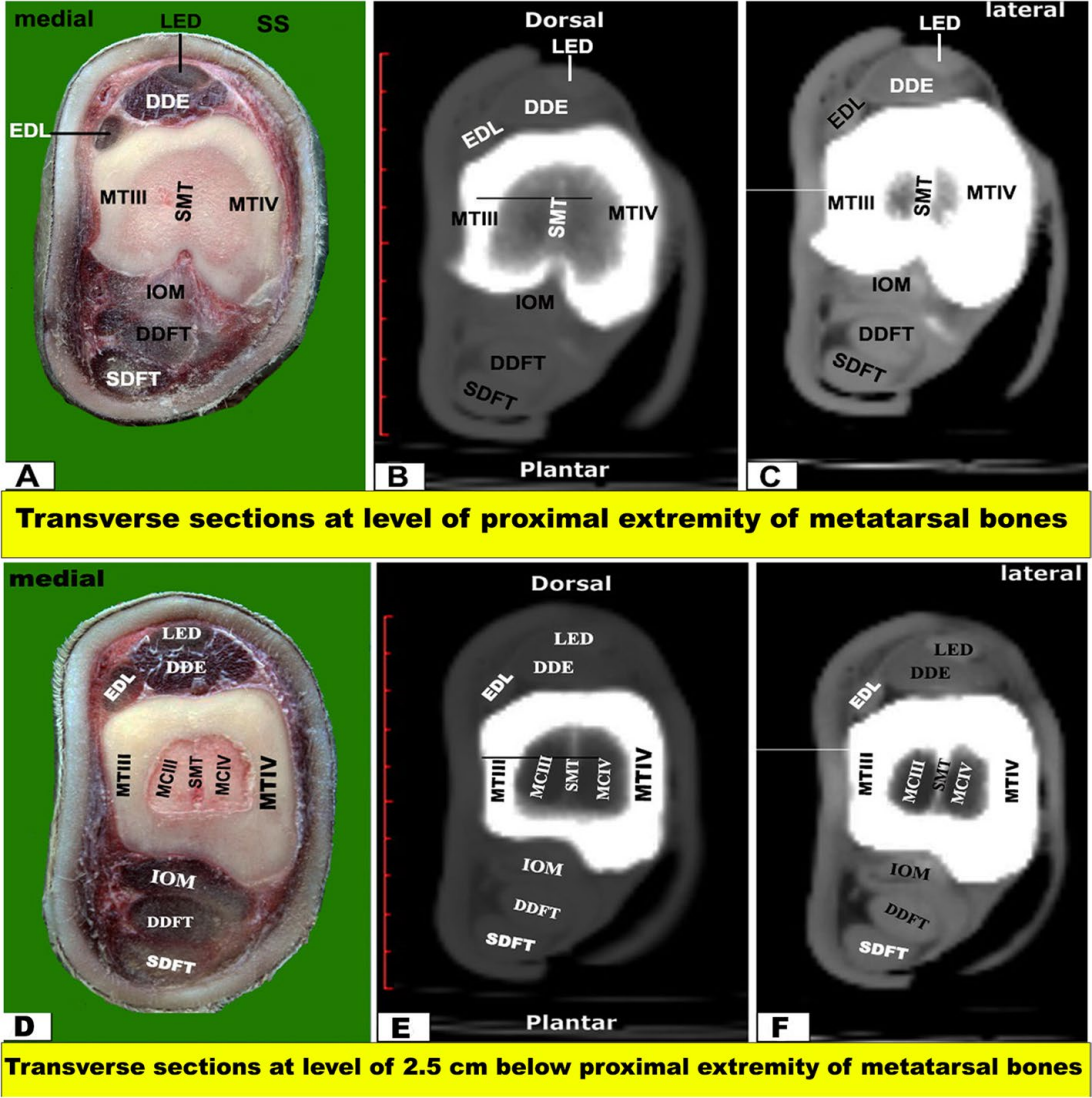
Transverse sectional anatomy of the pes region of Zebu bulls. Views **A** and **D** Gross and Views **B**–**C** and **E**–**F** CT sections. Views **A**–**C** represent the transverse sections at the level of the proximal extremity of the metatarsal bones (gross, bone windows, and soft windows). Views **D**-**F** represent the transverse sections at a level of 2.5 cm below the proximal extremity of the metatarsal bones (gross, bone windows, and soft windows). To show the (DDE) Deep digital extensor muscle; (EDL) Extensor digital lateral muscle; (IOM) Interosseous muscle; (DDFT) Deep digital flexor tendon; (LED) Long extensor digital muscle; (MT III) metatarsal bone third; (MT IV) metatarsal bone fourth; (SDFT) superficial digital flexor tendon; (SMT) septum between third and fourth metatarsal bones; (MCIII) the marrow cavity of the third metatarsal; (MCIV) the marrow cavity of the fourth metatarsal

**Fig. 5:**
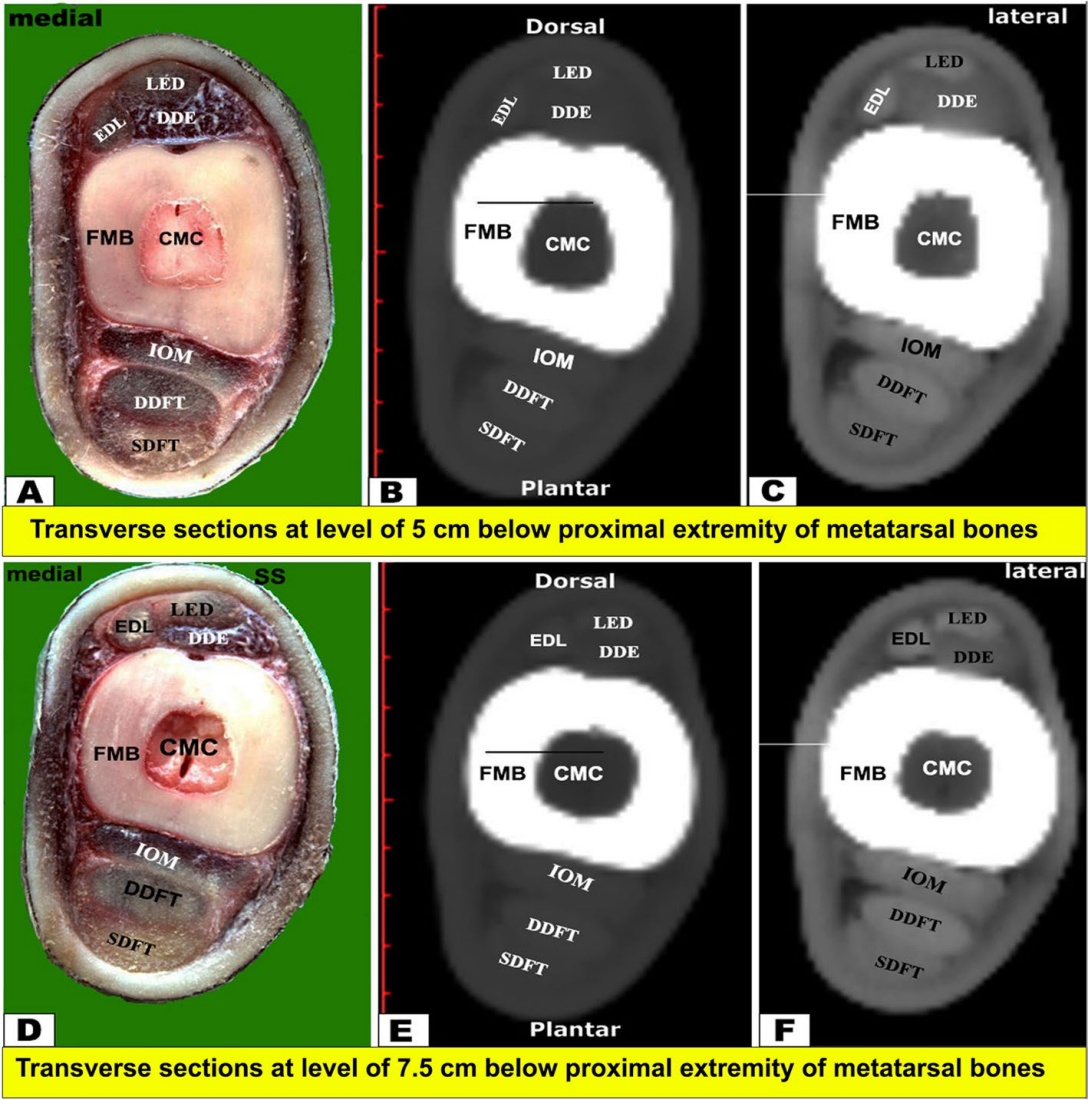
Transverse sectional anatomy of the pes region of Zebu bulls. Views **A** and **D** Gross and Views **B**-**C** and **E**–**F** CT sections. Views **A**–**C** represent the transverse sections at a level of 5 cm below the proximal extremity of the metatarsal bones (gross, bone windows, and soft windows). Views **D**-**F** represent the transverse sections at a level of 7.5 cm below the proximal extremity of the metatarsal bones (gross, bone windows, and soft windows). To show the (CMC) central marrow cavity; (DDE) deep digital extensor muscle; (DDFT) deep digital flexor tendon; (EDL) extensor digital lateral muscle; (FMB) fused metatarsal bones; (IOM) interosseous muscle; (LED) long extensor digital muscle; (SDFT) superficial digital flexor tendon

**Fig. 6:**
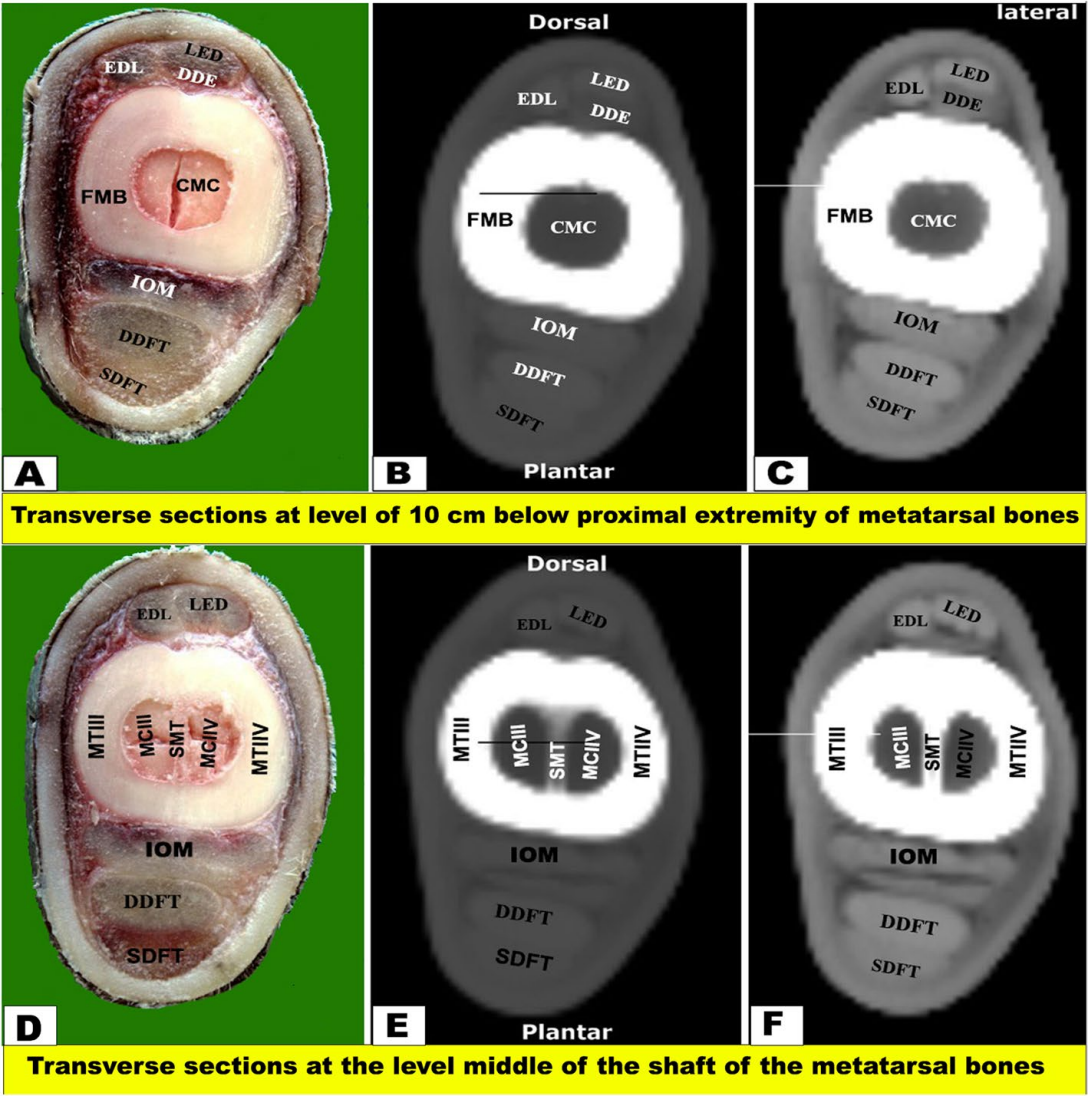
Transverse sectional anatomy of the pes region of Zebu bulls. Views **A** and **D** Gross and Views **B**-**C** and **E**–**F** CT sections. Views **A**-**C** represent the transverse sections at a level of 10 cm below the proximal extremity of the metatarsal bones (gross, bone windows, and soft windows). Views **D**-**F** represent the transverse sections at the level of the middle of the shaft of the metatarsal bones (gross, bone windows, and soft windows). To show the (CMC) central marrow cavity; (DDE) deep digital extensor muscle; (DDFT) deep digital flexor tendon; (EDL) extensor digital lateral muscle; (FMB) fused metatarsal bones; (IOM) Interosseous muscle; (LED) Long extensor digital muscle; (SDFT) Superficial digital flexor tendon; (MCIII) Marrow cavity of the third metatarsal; (MCIV) Marrow cavity of the fourth metatarsal; (MT III) Metatarsal bone third; (MT IV) Metatarsal bone fourth; (SMT) Septum between the third and fourth metatarsal bones

**Fig. 7:**
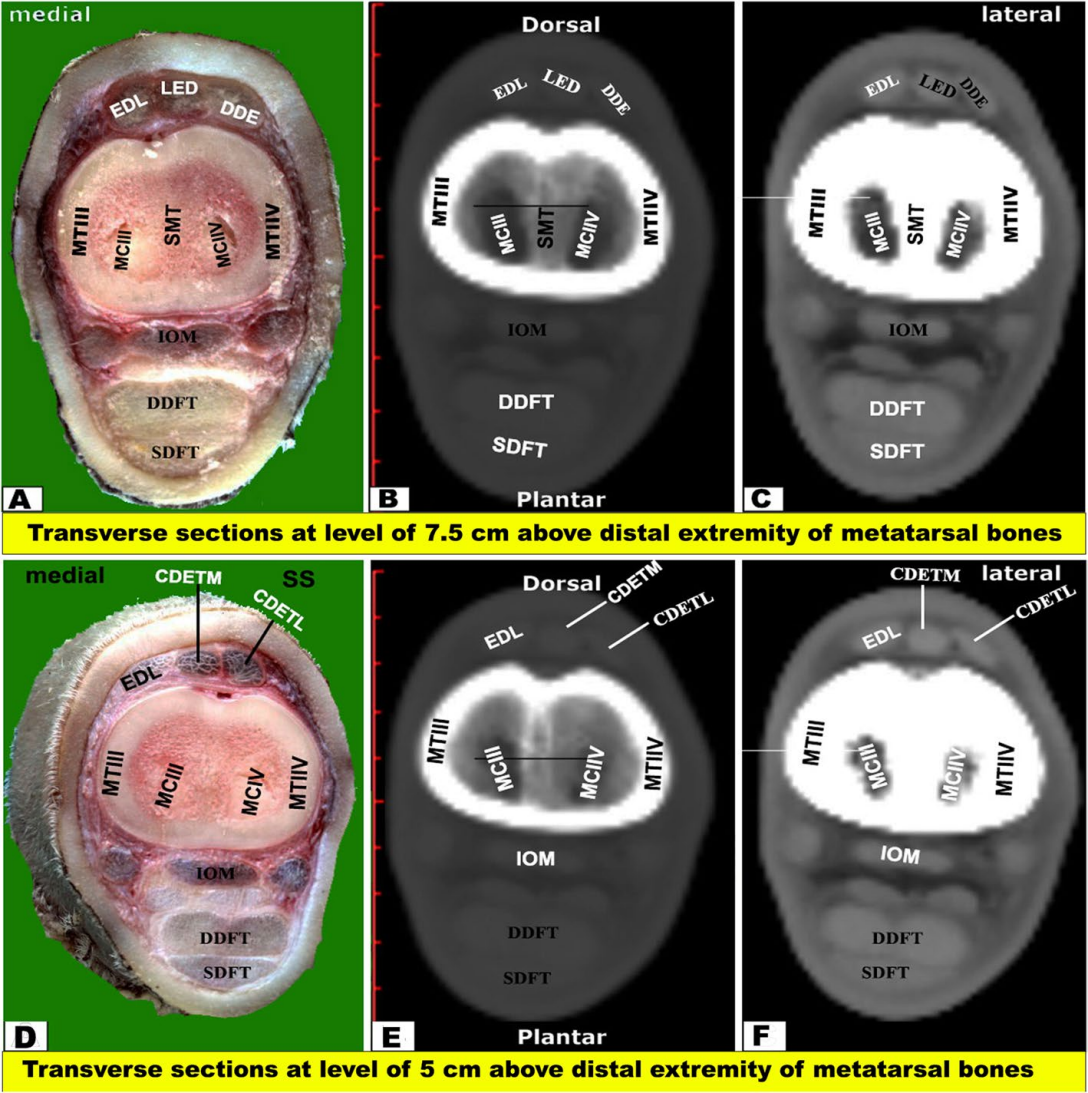
Transverse sectional anatomy of the pes region of Zebu bulls. Views **A** and **D** Gross and Views **B**-**C** and **E**–**F** CT sections. Views **A**–**C** represent the transverse sections at a level of 7.5 cm above the distal extremity of the metatarsal bones (gross, bone windows, and soft windows). Views **D**-**F** represent the transverse sections at a level of 5 cm above the distal extremity of the metatarsal bones (gross, bone windows, and soft windows). To show the (CDETL) Common digital extensor tendon (lateral part); (CDETM) Common digital extensor tendon (medial part); (DDFT) Deep digital flexor tendon; (EDL) Extensor digital lateral muscle; (IOM) interosseous muscle; (MCIII) marrow cavity of the third metatarsal; (MCIV) marrow cavity of the fourth metatarsal; (MT III) metatarsal bone third; (MT IV) metatarsal bone fourth; (SDFT) superficial digital flexor tendon; (LED) long extensor digital muscle; (SMT) septum between the third and fourth metatarsal bones

**Fig. 8:**
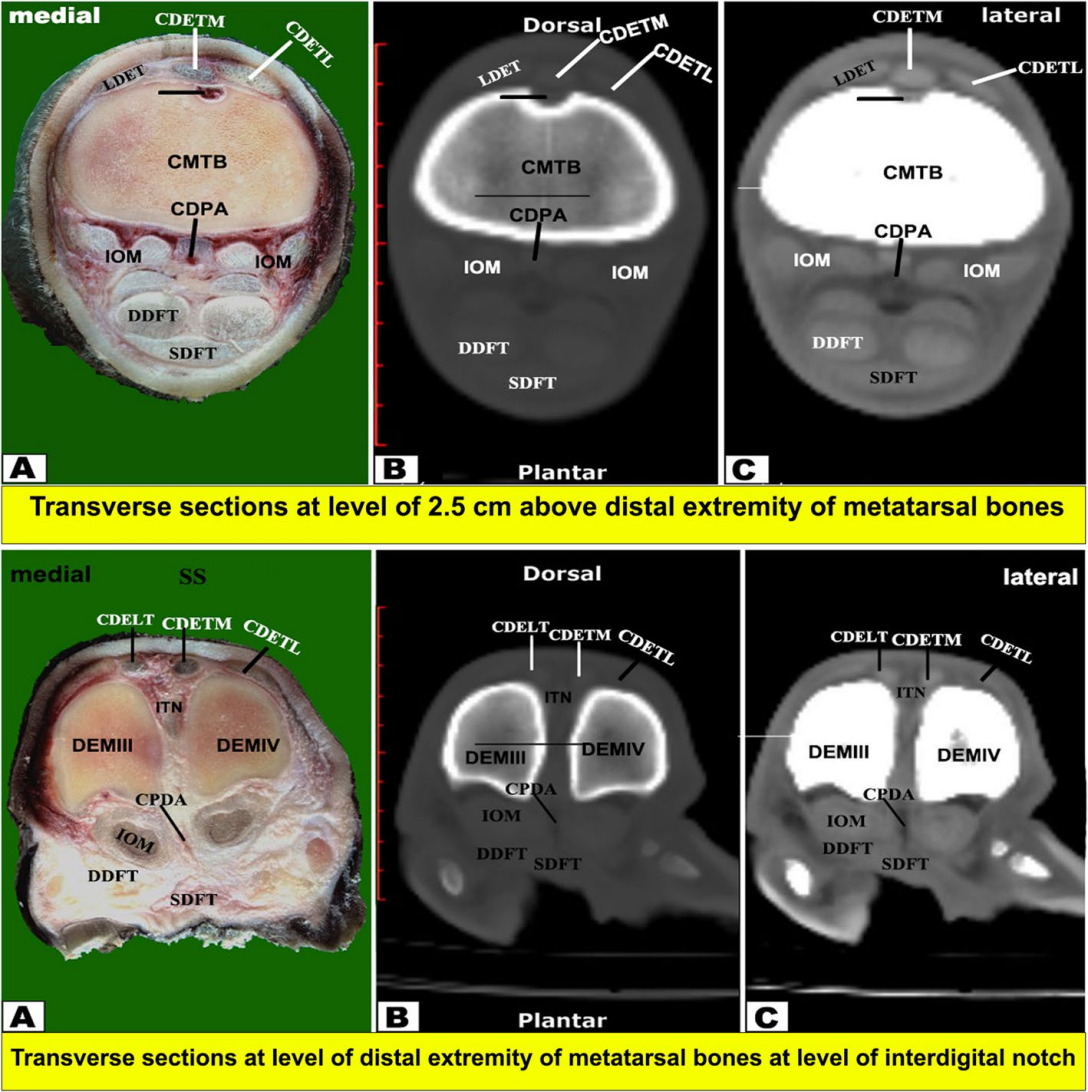
Transverse sectional-anatomy of the pes region of Zebu bulls. Views **A** and **D** Gross and Views **B**-**C** and **E**–**F** CT sections. Views **A**-**C** represent the transverse sections at the level of 2.5 cm above the distal extremity of the metatarsal bones (Gross, bone windows, and soft windows). Views **D**-**F** represent the transverse sections at the level of the interdigital notch (Gross, bone windows, and soft windows). To show the (CDETL) Common digital extensor tendon (lateral part); (CDETM) Common digital extensor tendon (medial part); (CPDA) Common planter digital artery (DDFT); Deep digital flexor tendon; (IOM) Interosseous muscle; (LDET) Lateral digital extensor tendon; (SDFT) Superficial digital flexor tendon; (DEMIII) Distal extremity of the third metatarsal; (DEMIV) Distal extremity of the fourth metatarsal; (ITN) Interdigital notch

**Fig. 9:**
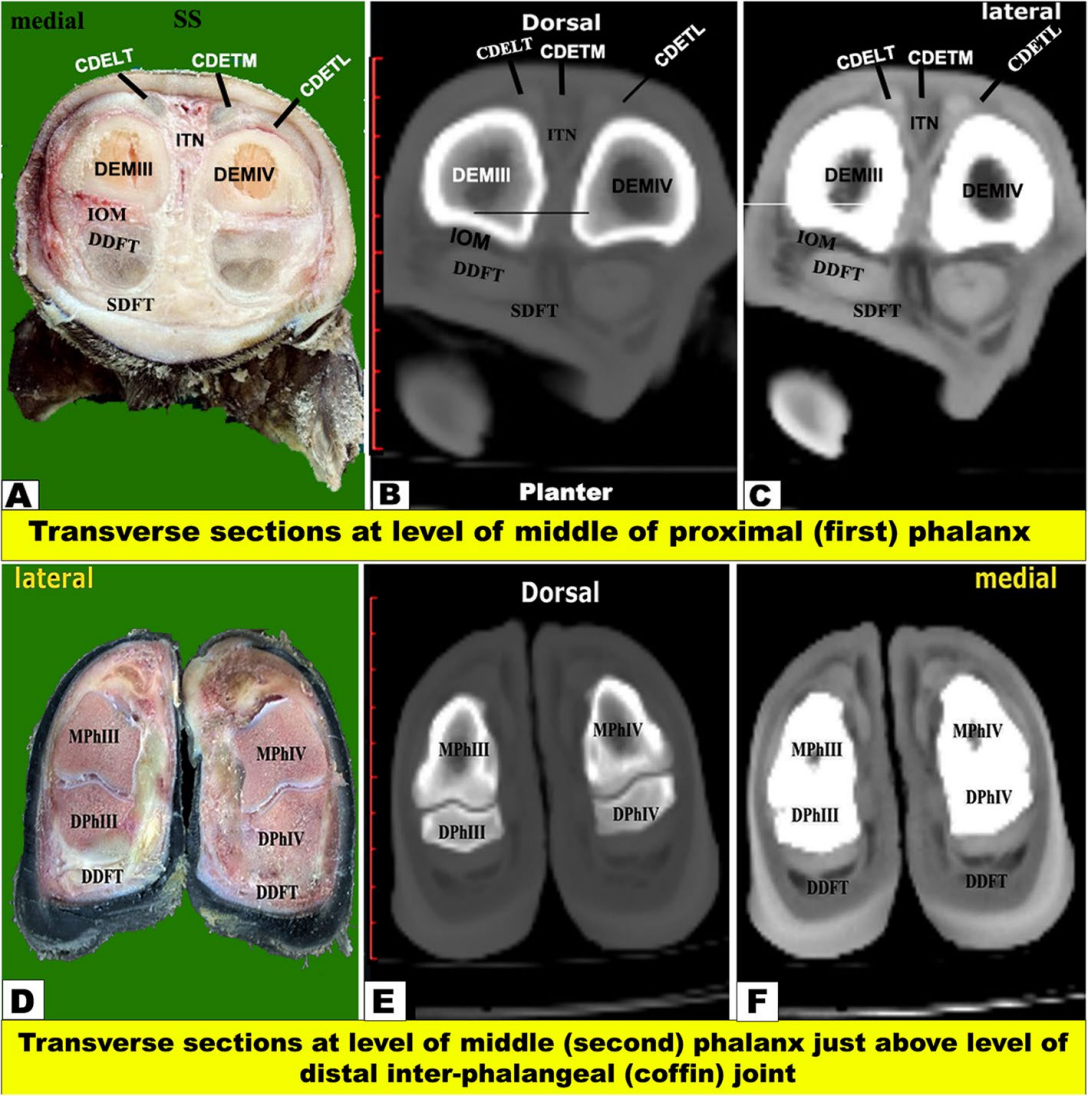
Transverse sectional-anatomy of the pes region of Zebu bulls. Views **A** and **D** Gross and Views **B**-**C** and **E**–**F** CT sections. Views **A**–**C** represent the transverse sections at the level of the middle of the proximal (first) phalanx (Gross, bone windows, and soft windows). Views **D**-**F** represent the transverse sections at the level of the distal inter-phalangeal (coffin) joint (Gross, bone windows, and soft windows). To show the (CDETL) common digital extensor tendon (lateral part); (CDETM) common digital extensor tendon (medial part); (DDFT) deep digital flexor tendon; (ITN) interdigital notch; (PPh) proximal Phalanx; (SDFT) superficial digital flexor tendon; (Mph) middle Phalanx; (DPh) distal phalanx

**Fig. 10:**
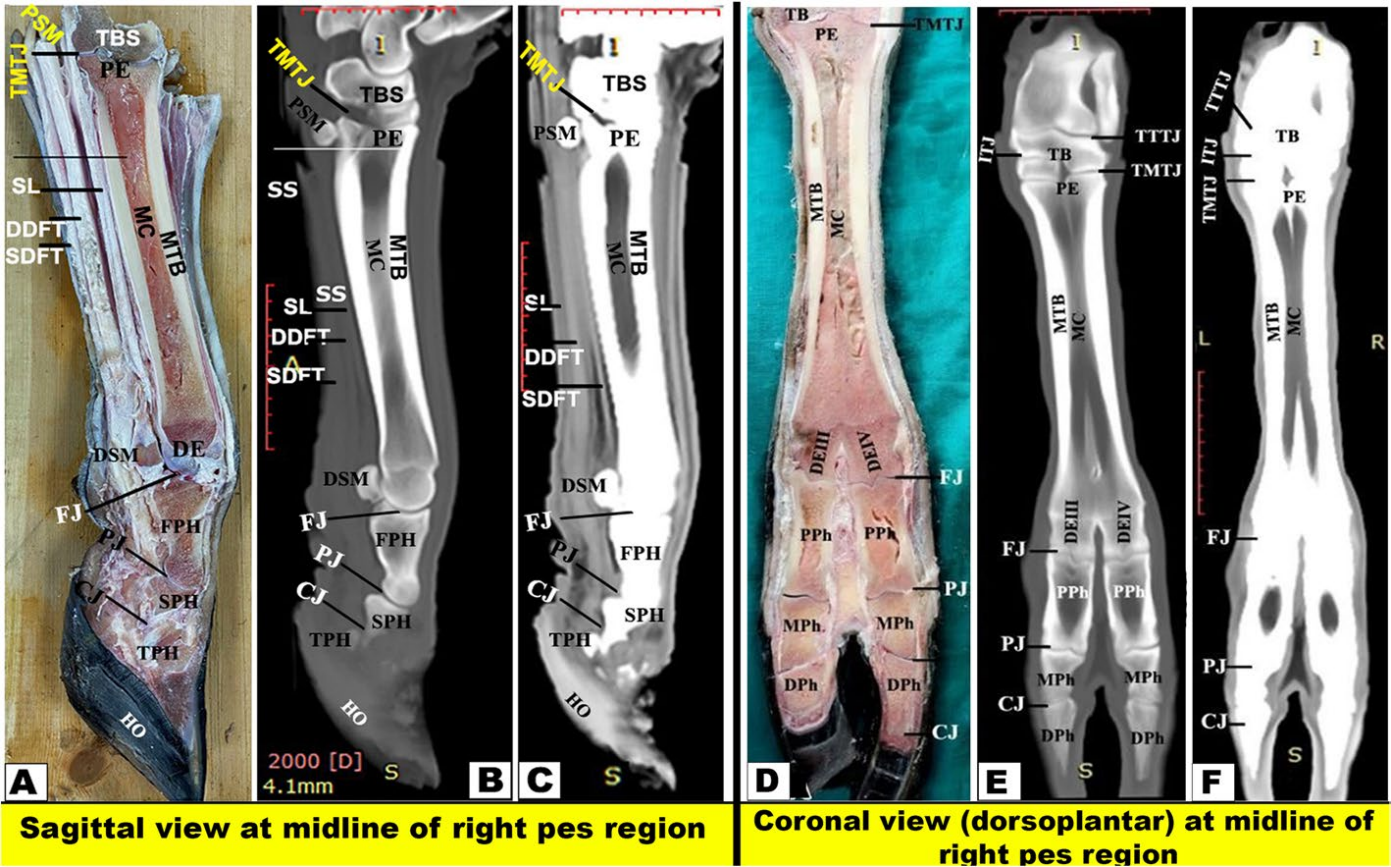
Sectional-anatomy of the pes region of Zebu bulls. Views **A** and **D** Gross and Views **B**-**C** and **E**–**F** CT sections. Views **A**-**C** represent the sagittal view at the midline of the right pes region at the level of the middle of the proximal (first) phalanx (Gross, bone windows, and soft windows). Views **D**-**F** represent the Coronal view (dorsoplantar) at the midline of the right pes region (Gross, bone windows, and soft windows). Notice the clear observation of the marrow cavity of the metatarsal and proximal phalanx. To show the articulations between the metatarsal bone (MTB), the proximal sesamoid (PE), the proximal, middle; and distal phalanges; and the arrangement of the flexor tendons in the plantar aspect, the soft window **(**sagittal view) and the level of the extension of the proximal and distal osseous septa **(**Coronal view) were shown. (CJ) Coffin Joint; (DDFT) Deep Digital Flexor Tendon; (DE) Distal Extremity; (DEMIII) Distal Extremity of Third Metatarsal; (DEMIV) Distal Extremity of Fourth Metatarsal; (DEMIII) Distal Extremity of third Metatarsal; (DPh) Distal Phalanx; (FJ) Fetlock Joint; (IOM) Interosseous Muscle; (MC) Marrow Cavity; (Mph) Middle Phalanx; (PJ) Patern Joint; (SDFT) Superficial Digital Flexor Tendon; (TMJ) Tarsometatarsal Joint

The transverse CT images from (Figs. 3, 4, 5, 6, 7, 8, and 9) start proximally at the level of the tibiotarsus joint and continue in a row distalward till the middle of the middle phalanx, i.e., just above the distal phalangeal joint. The average number of slices obtained was 300, with a 0.5 mm slice thickness. The sagittal CT images from **(**Fig. 10A-C), which were selected from about 50 slices with a 0.6 mm thickness starting from the medial towards the lateral aspect of the pes. The coronal CT images were selected from 60 CT views with a 0.6 mm thickness **(**Fig. 10D-F). In general, the soft window images allowed identification of the most clinically important soft tissue structures, including extensor tendons, superficial and deep digital flexor tendons, the digital cushion, collateral and sesamoidean ligaments, and the joint capsules of the digital joints **(**Figs. 3, 4, 5, 6, 7, 8 and 9**)**.

#### Sectioning of the frozen specimens

Two frozen specimens were placed on a table with an electric band saw, and 14 serial transverse sections were cut starting from the tibiotarsus joint and continuing distally until about the level just above the coffin joint (Fig. 1A) and from (Figs. 3, 4, 5, 6, 7, 8, and 9). Another two specimens were cut in sagittal sections at about the midline of the specimen (i.e., dorsoplantar axis) (Fig. 1B). One coronal section was obtained from another two specimens (Fig. 2), at about the middle. Attempts were made to slice each specimen exactly according to the chosen axis.

The collected slices for each region were numbered and gently cleaned with a light toothbrush soaked in water, left for a while to dry, and then photographed using Canon (EOS 2000D 18–55 IS, 24.1 MP, DSLR digital camera), and the selected surfaces were faced towards the camera, i.e., proximal surfaces of transverse sections (Figs. 3A, D; 4A, D; 5A, D; 6A, D; 7A, D; 8A, D; 9A-D), right surfaces of sagittal sections (Fig. 10A), and dorsal surfaces of the coronal sections **(**Fig. 10D). Important anatomical structures were identified on each section and correlated to their analogous structures on corresponding soft and bone CT window images. Closely matched CT and anatomical views were selected, labelled, and presented in **(**Figs. 3B-C, E-F; 4B-C, E-F; 5B-C, E-F; 6B-C, E-F; 7B-C, E-F; 8B-C, E-F; 9B-C, E-F) for transverse views, (Fig. 10B-C) for sagittal views, and (Fig. 10E-F) for coronal views.

#### Gross anatomy

Two fresh distal limbs were fixed in 10% formalin and then carefully dissected for studying the morphological features and used as references for the anatomic structures of the pes region, including muscles, tendons, ligaments, and vessels. Another two fresh specimens were macerated and prepared as the skeletal structures; metatarsus, phalanges, and sesamoid bones.

### Statistical analysis

The normality of the data was assessed, and an independent t-test was conducted to statistically analyze the morphometric values of the tarsal region and pes region (metatarsal, proximal, middle, and distal phalanges) of the Zebu bulls’ hind limb (cm) by using the SPSS Statistics for Windows, version 23.0. Armonk, NY: IBM Corp. The data are presented as means ± standard deviations and difference was declared as-significant when (*p* < 0.05) and highly significant when (*p* < 0.01).

## Results

### Radiography (X-ray)

**Fig. 11:**
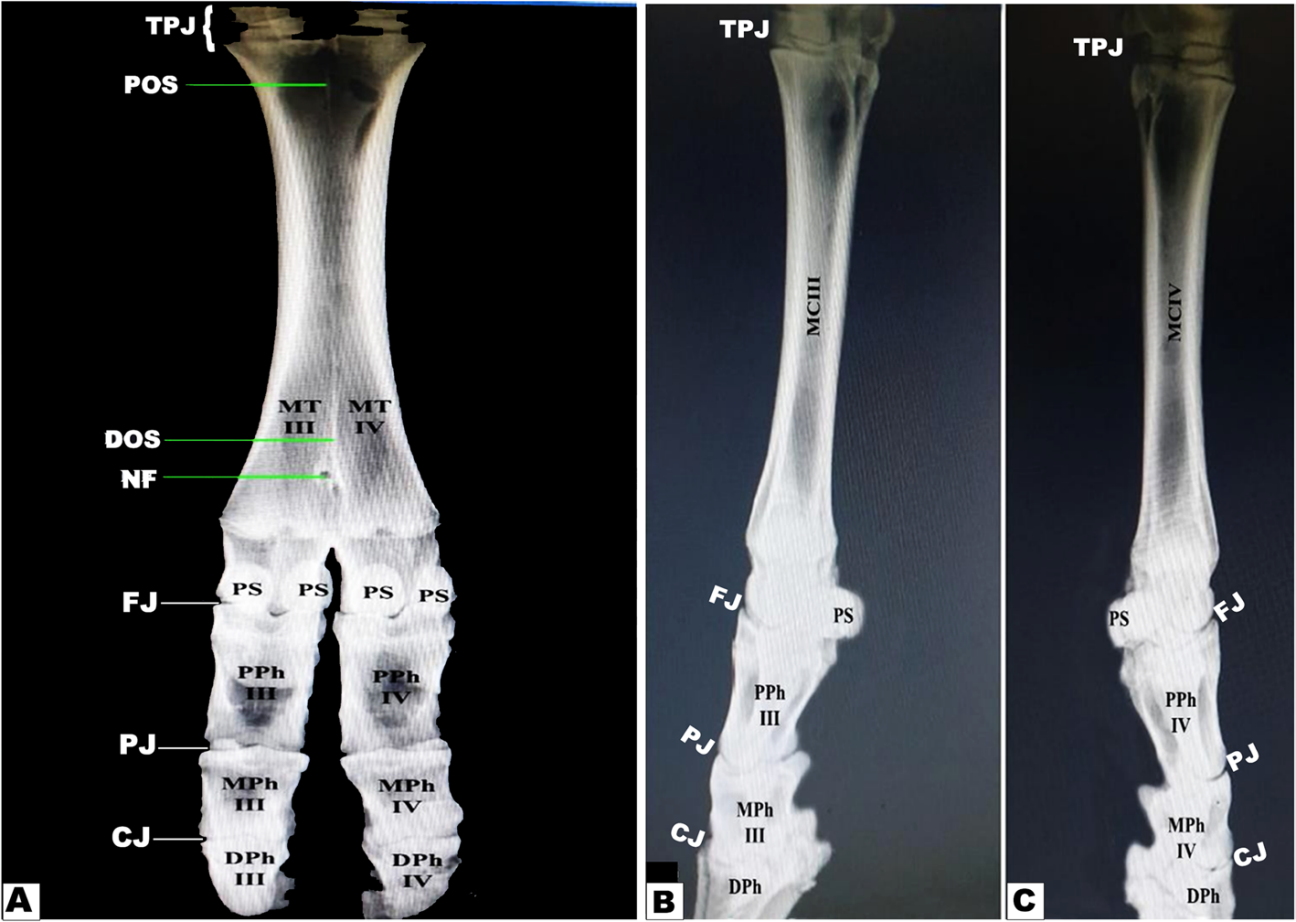
Radiographic images of the pes region of Zebu bulls. View **A** represents the dorsoplantar, and (View **B**) is a lateral radiograph of the normal structure of the skeletal components, showing the nutrient foramen (NF); the proximal (POS) and distal (DOS) osseus septa; the proximal sesamoid (Ps); the proximal phalanx (PPh); the middle phalanx (Mph); and the distal phalanx (DPh); the Marrow cavity of the third metatarsal (MCIII); and the Marrow cavity of the fourth metatarsal (MCIV)

The dorsoplantar and mediolateral radiographs of the investigated specimens showed normal morphology of the musculoskeletal and soft structural components of the distal limb of the Zebu bulls. All bones and cartilage presented high contrast compared to soft tissue structures. Dorsoplantar (Fig. 11A) and lateral (Fig. 11B–C) radiographic views permitted a definitive visualization of the skeleton, including three segments from proximal to distal: tarsal bones, metatarsal bones, and phalanges. Five tarsal bones were arranged in three rows: the proximal row comprised the talus and calcaneus; the middle row included the central bone; and the distal row was formed of three fused bones: the 2nd, 3rd, and 4th tarsal bones.

The III and IV large metatarsal bones were fused along their most parts; however, radiopaque proximal and distal osseous septums were clearly observed projecting into the medullary cavity from the proximal and distal extremities, respectively (Fig. 11A). The distal extremity of the large bone was divided distally by an intertrochlear notch into the medial and lateral trochlea. The medial and lateral trochlea articulated with the proximal phalanx at the metatarsophalangeal (MPJ) or fetlock joint, forming the bases of the III and IV digits, respectively. In addition, the nutrient foramen was clearly observed just above the interdigital notch of the metatarsal bone (Fig. 11). Each digit comprised proximal, middle, and distal phalanges in addition to proximal and distal sesamoid bones. Two sesamoid bones were superimposed over each trochlea; the middle bone was larger than the lateral one. The distal sesamoid was shown at the plantar aspect of the third phalanx.

### Computed tomographical anatomic references of the pes region

The CT soft tissue window outlined the soft tissues, including the suspensory ligament, extensor, superficial, and deep digital flexor tendon, digital scutum, collateral ligaments, and joint cavity. The CT bone window permitted the recognition of the pes regions' bony parts, including those of the articular surface and bone marrow cavities. All of the surfaces and structures of the digit's bony components, including the articular surface, were clearly explained by the three-dimensional volume rendering of the CT scan. Different patterns of the compact, spongy, subcortical bones and marrow cavities of the bones of the digits were visible in the processed 3DVR of CT images.

#### Sectional anatomy and computed tomography scans

In the present study, we found CT to be an excellent modality for providing anatomical images of the skeletal and soft tissue structures of the distal limb. In general, CT scans had higher density for the bones, and the entire image had excellent delineation between the compact substance and medullary cavity of the bones, and the trabecular pattern of the cancellous bone was clearly shown. All bones of the pes region, including the tarsal bones, extremities and diaphysis of the metatarsus, proximal, middle, and distal phalanges, as well as the proximal and distal sesamoid bones, were seen on the transverse-, sagittal-, and coronal-plane images.

#### Extensor tendons

At the level of the tibiotarsal joint, the crural extensor retinaculum (CER) and metatarsal extensor retinaculum (MER) extended on the dorsal aspect of the tarsus between the first tarsal bone (FTB) on the medial aspect and the caudolateral aspect of the calcaneus and talus on the lateral aspect (Fig. 3). These retinacula enclosed the tendons of extensor muscles of the tarsus, passing along the dorsal and medial aspects, including fibularis tertius (FTT) and cranial tibial (CTT), and the dorsal and lateral aspects, including fibularis longus (FLT), long digital extensor (LED), short digital extensor (DEMS), medial and lateral limbs of the long digital extensor muscle (EDLM & EDLL, respectively), lateral digital extensor (DELT/EDL), deep digital extensor (DDE), and lateral part of the digital extensor (DETL).

The CTT lied on the dorsal surface of the tibia deep to the fibularis tertius tendon, and it was inserted in the first tarsal bone, fused second and third tarsal bones, and the proximomedial surface of the large metatarsal bone. Just below the level of the tarsal joint, only the tendons of LED and DDE continued their courses on the dorsal aspect of the metatarsus, while EDL continued on the lateral aspect **(**Figs. 4, 5, 6 and 7A-C**)**. Each tendon was completely enclosed by its own tendon sheath. The tendon of DEMS was blended with that of LED. At about 7.5 cm above the distal extremity of the metatarsal bones, LED detached medial (deep) and lateral (superficial) tendons (DETM and DETL, respectively) **(**Fig. 7D-F**)**.

#### Flexor tendons

On the plantar aspect of the metatarsal bones, the interosseous muscle (IOM) appeared conspicuous and appeared as a flattened cord. The superficial digital flexor tendon (SDFT) and deep digital flexor tendon (DDFT) were also readily identifiable on all planes of CT images and sectional anatomy (Figs. 3, 4, 5, 6, 7, 8 and 9). SDFT and DDFT were smoothly marginalized along the plantar aspect of the IOM and the fused metatarsal bones. Each tendon was surrounded by a small rim representing its tendon sheath and bifurcated into medial and lateral limbs proximal to the fetlock joint. Each limb of the SDFT received a connecting band from the IOM, forming a sleeve around the corresponding band of the DDFT proximal to the metatarsophalangeal joint (Figs. 7D-F, 8 and 9A-C). Distal to the proximal sesamoid bones and prior to its insertion in the base of the middle phalanx, SDFT gained a position deeper than that of DDFT (Fig. 8A-C).

### 3D render volume reconstruction of CT

**Fig. 12:**
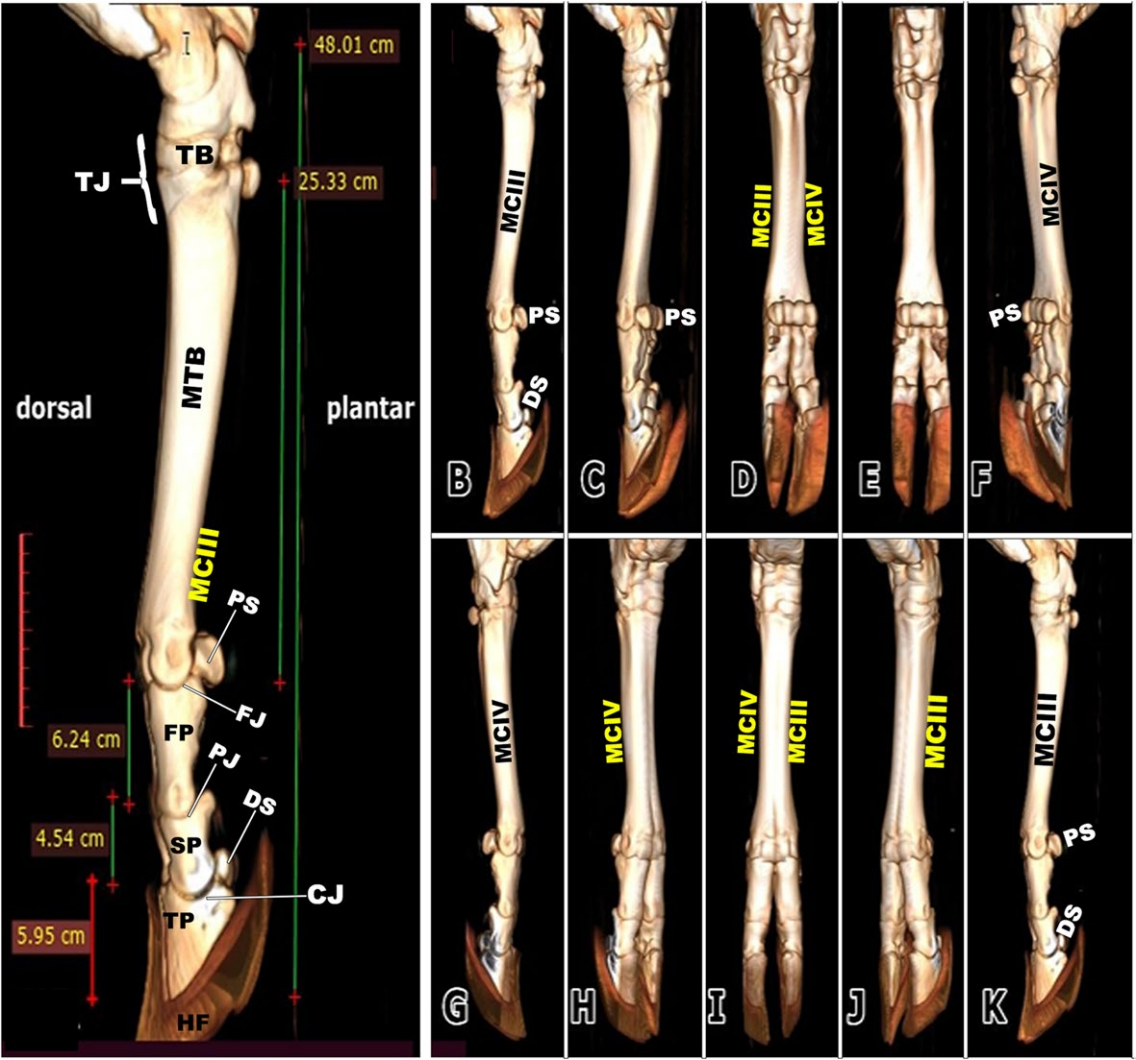
3D render volume reconstruction of the left pes region of the zebu hind limb showed the dorsal and plantar views (**A**) and the reconstruction pattern step by step (**B**-**K**) illustrated the different orientations of the tarsal, metatarsal, sesamoid, and phalanges to get a complete diagnostic image of the normal structure of the skeletal components and their length average. To show (CJ) Coffin joint; (PJ) Pastern joint; (FJ) Fetlock joint; (TB) tarsal bones; (TJ) tarsal joint; (MTB) Metatarsal bone; (MCIII) third metatarsal bone; (MCIV) fourth metatarsal bone; (FP) Proximal Phalanx; (SP) second Phalanx; (TP) third Phalanx; (PS) proximal Sesamoid bone; (DS) distal Sesamoid bone; (HF) hoof

A 3D render volume reconstruction of a CT scan provided numerous 360-degree photos that provided good confirmation of the pes region structures as well as 3D explanations of the details of the digit bones. The present study used imaging techniques to describe the bones of the pes region in Zebu bulls. The 3D CT produces detailed images of the bones of the pes region. Reconstruction of the 3D render volume was performed on the various CT oblique dorsal and plantar views of the pes regions. This improved our ability to see the bones of the digit, metacarpal, and carpal regions from all angles (Fig. 12). The bones of the same leg's medial and lateral sides could be seen in the 3D reconstruction of the bone, and the sizes of the bones in the right and left limbs were remarkably similar.

Each pes region had two digits (left and right) that were located distal to the distal extremities of the third and fourth metatarsals. Each digit is formed from the proximal, middle, and distal phalanges. The longest digit bone was a nearly semi-cylindrical-shaped first phalanx that had a compressed proximal articular surface (Fig. 12) and had two facets that were divided by an intermediate sagittal notch. The sesamoid bones and these facets have articulated (Fig. 12). This extremity forms the fetlock joint (Fig. 12) with the distal extremities of the 3rd and 4th metatarsal bones (Fig. 12). The proximal extremity also has rough areas on the sides in addition to the transverse ridge on the axial side (Fig. 12).

The first phalanx's strongly convex distal articular surface extended proximally to the dorsal side and further to the plantar side. The second phalanx was compressed dorsoplantarly, convex dorsally, and flattened plantarly. It was roughly two-thirds the length of the first phalanx. Its proximal extremity had a concave articular surface and an elliptical shape. On the transverse ridge of the proximal extremity of the second phalanx, an extensor process could be seen (Fig. 12). Its distal articular surface was convex and extended on the dorsal and plantar surfaces, and its distal extremity featured depressions for ligament attachment on each side (Fig. 12). The third phalanx had the appearance of being wedge-shaped, with the apex facing dorsally and the articular surface being somewhat flat (Fig. 12). The distal extremities of the third and fourth metatarsal bones showed characteristics like the trochlea, intertrochlear notch, tubercle, and depressions on the axial and abaxial aspects (Fig. 12).

### Statistical analysis

The means of the morphometric values of the tarsal region and pes region (metatarsal, proximal, middle, and distal phalanges) of the Zebu bulls hind limb was assessed, and an independent t-test was conducted to statistically analyze their morphometric values using the SPSS Statistics, as described in the (Table 1). The measurements of the pes region and its structures (Table 1) showed that the right side had a minor increase in values compared to the left side, and the lateral sides had higher values than the medial sides.

**Table 1:** The means of the morphometric values of the tarsal region and pes region (metatarsal, proximal, middle, and distal phalanges) of the Zebu bulls hind limb by cm

Anatomical structures	Right side	Left side
Tarsal region	5.95 ± 0.22	5.1 ± 0.12
Pes region	42.06 ± 2.7	37.5 ± 2.1
Metatarsal region	25.33 ± 1.1	21.56 ± 1.9
Phalangeal region	16.73 ± 0.81	13.34 ± 0.9
First phalanx region	6.24 ± 0.32	5.1 ± 0.34
Second phalanx region	4.54 ± 0.24	3.1 ± 0.4
Third phalanx region	5.95 ± 0.34	4.6 ± 0.54

Means within the same column under the same category carry different superscripts a highly significantly different (*p* < 0.01)

## Discussion

The Zebu cattle (*Bos Taurus indicus*) were characterized by a fatty hump on their shoulder, which they adapted to living in high temperatures as they adapted for tropical environments and were bred mainly for meat with very little milk production [1, 2]. Our study was prepared to provide a complete gross, cross-sectional, and computed tomography (CT) image of the pes region of Zebu bulls. The clinical importance of our study is due to the fact that in ruminants, diseases of the metatarsus and digits are common, making knowledge of the normal structure necessary in order to spot variations in the afflicted animal. Numerous studies have been conducted in this area to better understand the pathophysiology The surgeons and veterinarian students have the ability to apply our obtained data for diagnosing and treating specific PES disorders in Zebu cattle. This application of data can greatly enhance the accuracy and effectiveness of their medical interventions, leading to improved health outcomes for Zebu cattle. Additionally, it empowers these students to contribute to ongoing research and advancements in the field of veterinary medicine. of bovine hoof and digit disorders because the economic significance of cattle lameness is well acknowledged [37]. According to AR Raji, K Sardari and H Mohammadi [18] in the bovine digits and A Al Aiyan, FC King, A Aldarwich, U Kishore and T Shawaf [12] in the one-humped camel digits, a complicated structure comprises joints, ligaments, and tendons. Traditional anatomic atlases are unable to offer the range of perspectives and level of information needed for contemporary diagnostic and surgical approaches [13, 38].

New diagnostic anatomical techniques, such as computed tomography (CT), magnetic resonance imaging (MRI), and other techniques, offer high-contrast, high-resolution images for veterinary medicine diagnosis [12, 20, 39]. CT provides high-contrast images of various body parts, such as bones, joints, and soft tissue components of the tarsus, which can be compared with bone models and dissected specimens to aid in the accurate identification of specific structures [12, 40]. Despite its limited use in ruminants, radiography remains a standard diagnostic procedure in veterinary medicine [12, 13, 41]. Ultrasonography allows for the visualization of tendons and ligaments but has limited usefulness for soft tissue examination [42]. Additionally, ultrasonography requires individual photography, making it difficult to examine the entire finger cross-sectional. However, new diagnostic anatomical techniques, such as CT and MRI, offer valuable resources for veterinary medicine diagnosis. However, current methods like ultrasonography and radiography still have limitations in examining soft tissue in digits and hoofs. Further research is needed to fully utilize these advanced techniques in veterinary medicine [13, 43].

Computed tomography (CT) is a widely used imaging technique in human and veterinary medicine for investigating various diseases and improving the detection of animal disorders [12, 44]. Initially used in ruminants due to its limited accessibility and high costs, CT has since become a standard diagnostic procedure in veterinary medicine, but now computed tomography (CT) has become an accepted alternative imaging modality that far surpasses what can be provided by survey radiography and is used to improve the detection of animal disorders [12, 45]. New diagnostic anatomical techniques, such as CT and MRI, provide high-contrast, high-resolution images for describing and diagnosing all body parts as described in the one-humped camel digits [12]. However, accessibility has improved, which has increased the need for the use of this technique in all animal species [8, 9, 15, 18, 27, 40, 46]. Computed tomography offers improved spatial resolution and effective bone-soft tissue separation [12, 40, 47, 48] compared to traditional radiography, which requires a 10% physical density difference for visual detection [49]. However, its use in veterinary medicine is restricted due to cost and animal anesthesia requirements [47]. CT offers cross-sectional images with greater soft tissue distinction and no superimposition of surrounding structures, making it more accurate in diagnosing anomalies [18].

In the current investigation, it was described that the pes region of the zebu bull had three segments that were described as the following from proximal to distal: the tarsal bones, the metatarsal bones, and the phalanges. Moreover, there are five tarsal bones that are arranged in three rows as follows: the proximal row (talus and calcaneus); the middle row (central bone); and the distal row that is formed from three fused bones: the 2nd, 3rd, and 4th tarsal bones. Furthermore, the fused III and IV large metatarsal bones and a small metatarsal bone (II) on the medio-plantar aspect were readily visible. Similar observations about the number of tarsal bones and the metatarsal bones were reported in the cattle and camel [4, 5, 12, 50]. Anatomically, there are species variations in the number of the metatarsals between the different domesticated animal species, which reach five in number in humans, dogs, and ring-tailed lemurs [4–6, 51], four in pigs [4, 5, 52, 53], three in ruminants, equines, and pigs [4, 5]. In the same animals, the metatarsal bones are divided into large functional and small rudimentary bones. The ruminant’s species had two large functional and one small rudimentary bone, while the equine had only one large functional and two small rudimentary bones [4, 5].

The current work describes that the two large fused (III and IV) metatarsal bones of the examined zebu bull were completely fused except for the distal extremities of the two bones. However, there is incomplete fusion between these two large metatarsal bones, in which the distal fifth is separated in the camel [19]. The distal extremity is divided into two parts by the presence of the sagittal intercondyloid groove (cleft), similar to that reported by SC Yadav, S Joshi, R Mathur and OP Choudhary [54] in the chital (*Axis axis*) and D Raghavan [55] in the ox. Our results described that the distal extremity of the large bone was divided distally by the intertrochlear notch into the medial and lateral trochlea; additionally, the medial and lateral trochlea articulated with the proximal phalanx at the metatarsophalangeal or fetlock joint, forming the bases of the III and IV digits, respectively. The same results were reported for all domesticated large ruminant species [4, 5]. In cattle, the intertrochlear incisures is 3 cm in length and present only between the two trochleae, while in camels, its length is about 6 cm and extends beyond the trochlea proximally between the distal parts of the metatarsal III and IV shafts [50]. The distal metatarsal canal, which is present in cattle, is absent in camels [50]. Our results described that each digit of the examined zebu bull was formed of the proximal, middle, and distal phalanges in addition to the proximal and distal sesamoid bones. Two sesamoid were superimposed over each trochlea, the middle bone was larger than the lateral one. The same results were reported for all domesticated large ruminant species [4, 5]**.**

Our CT examinations provided excellent tomographic images of the Zebu distal limb's skeletal and soft tissue structures. The scans had higher bone density and excellent delineation between the cortex and medullary cavity. The trabecular pattern of the cancellous bone was clearly shown. All bone structures, including tarsal bones, extremities, metatarsus, phalanges, and sesamoid bones, were visible on transverse, sagittal, and coronal-plane images. Soft window images identified clinically important soft tissue structures, including extensor tendons, digital flexor tendons, digital cushion, collateral and sesamoidean ligaments, and joint capsules. The same results describing the clarity of the CT images were noted by [7–9, 19, 56]. The soft window images of the CT technique enable the identification of clinically important soft tissue structures, such as extensor tendons, digital flexor tendons, digital cushion, collateral ligaments, and joint capsules. These findings have been cited by various studies, emphasizing the importance of these images in detecting and treating various soft tissue conditions [7–9, 15, 19, 20, 56]. The New Imaging technique aids in diagnosing lameness in dairy animals under modern housing conditions, attributed to reduced physical activity [56, 57]. Lameness in buffalo hind feet is common in veterinary practice, affecting primary or secondary limbs [58, 59]**.**

Zebu bulls exhibit similar anatomical and morphometric features to other cattle species but have distinct differences. The more clear variation is the metatarsal bones' length from proximal to dorsal boundary, and the length from the proximal extremity to the dorsal boundary of the metatarsal groove differs significantly from other species [3–6]. According to our research, the large metatarsal's distal extremity is separated distally into two portions called the medial and lateral trochlea by an intertrochlear notch that is about 3 cm long and 2.5 cm wide. The medial and lateral trochlea articulated with the proximal phalanx at the metatarsophalangeal joint, forming the bases of the III and IV digits. Similar results about the division of the distal extremity of the large metatarsal bone and their articulation with the first phalanx were reported by [4, 5, 50].

The current work reported that the cortical thickness of the metatarsal bone of the examined zebu cattle was equal on both sides at the level of the midportion of the shaft, forming about 1.62 cm, and thinning occurred gradually towards the extremities. Thickening of the dorsal and plantar cortices was almost equal in sagittal CT views. Moreover, in the dorsoplantar views, the cortical thickness is equal on both sides at the level of the midportion of the bone, and thinning occurs gradually towards both ends. Our study described that the bony septum in the shaft of the metatarsal of the zebu bull divided the medullary cavity between the two fused large metatarsal bones, was observed only in the proximal distal half, and disappeared in the middle part. While in buffalo, this septum was completely absent, in camels and cattle, this septum was complete along the fused part of the two bones and completely divided the medullary cavity [19, 50]. In the examined zebu cattle, the proximal and distal extremities of the two fused large metatarsal bones had a vertical bony septum, similar to that described by A El‐Shafey and A Kassab [19] in buffalo and camels. In sheep and goats, H Bahgat [60] described that the medullary cavity of the fused third and fourth metacarpal bones was divided internally by a vertical bony septum at the proximal and distal extremities. In sheep and goats, H Bahgat [60] found that on the dorsal aspect of the fused third and fourth metacarpal bones and proximal and middle phalanges, the digital extensor tendons were differentiated in the cross-sectional anatomy only when the fascia dorsalis manus was dissected. In the one-humped camel, A El‐Shafey and A Kassab [19] reported that the medullary cavity of the fused third and fourth metacarpal bones was divided internally by a vertical bony septum, which was a complete septum in the camel, while it is complete at the proximal and distal extremities in the buffalo, while it is small and incomplete in the main part of the fused shaft of the metacarpal bones in the buffalo.

Our CT examination showed the adjacent extensor tendons as a transverse narrow strap with undifferentiated outlines on the dorsal aspect of fused metacarpal bones, proximal phalanges, and middle phalanges, and the adjacent flexor tendons as a roughly rounded mass with undifferentiated outlines on the palmar aspect of fused metacarpal bones, proximal phalanges, and middle phalanges. Similar observations were reported in both camels and buffalo [19]. In the current coronal and transverse CT views, it is observed that there is an incomplete hyper-attenuated vertical and wedge-shaped septum between the III and IV metatarsal bones, referring to the incomplete fusion of the proximal and distal ends of these bones. The current investigation noted that the proximal septum was extended downward for about 7.85 cm away from the proximal extremity, while the distal septum was extended upward for about 10.10 cm away from the intertrochlear notch.

Anatomically, the metatarsal bones are long bones between the tarsal bones and the first phalanx, named by number from the medial to the lateral side. They are abbreviated as the first (I), second (II), third (III), fourth (IV), and fifth (V) metatarsal bones [4, 5]. Metatarsal bones vary among animal species, with humans and dogs having five, pigs having four, and ruminants and horses having three [4–6, 19, 50]. Domesticated animals' metatarsal bones are classified into large functional and small rudimentary types, with ruminants having two functional and one small rudimentary bone and horses having one large functional (III) and two small rudimentary (II and IV) metatarsal bones [4, 5]. Our study found that two sesamoid bones in zebu bulls were located plantar to each trochlea of the large metatarsal bone, with the middle bone being larger than the lateral one. These sesamoid bones appeared as a vertical pear-shaped structure with a 2.5 cm vertical length and a 1.6 cm width. In cattle, the proximal sesamoid bones are four in number, two for each trochlea, and in the dorsoplantar projection, the two sesamoid bones are superimposed over each trochlea; the middle bone is larger than the lateral one [50]. Moreover, they appear as a vertical pear-shaped structure with a 2.5 cm vertical length and 1.6 cm width (lateral bone), as described by [50].

The current work reported that at about the level of the tibiotarsal joint, the crural extensor retinaculum (CER), metatarsal extensor retinaculum (MER), and the extensor tendons were extended on the dorsal aspect of the tarsus between the first tarsal bone (FTB) on the medial aspect and the caudolateral aspect of the calcaneus and talus on the lateral aspect. These retinacula enclosed the extensor muscles of the tarsus, passing along the dorsal and medial aspects, including fibularis tertius (FTT) and cranial tibial (CTT), and the dorsal and lateral aspects, including fibularis longus (FLT), long digital extensor (LED), short digital extensor (DEMS), medial and lateral limbs of the long digital extensor muscle (EDLM & EDLL, respectively), lateral digital extensor (DELT/EEDL), deep digital extensor (DDE), and lateral part of the digital extensor (DETL). On the plantar aspect of the metatarsal bones, the interosseous muscle (IOM) appeared conspicuous and appeared as a flattened cord. The superficial digital flexor tendon (SDFT) and deep digital flexor tendon (DDFT) were also readily identifiable on all planes of CT images and sectional anatomy. At the level of the proximal extremity of the proximal phalanx, each superficial digital flexor tendon was seen forming a sleeve (*manica flexoria*) around the corresponding deep digital flexor tendon [56]. The lateral branch of the long digital extensor tendon maintained its course along the median part of the dorsal aspect of the limb, flanked by the medial branch of the same named tendon and the lateral digital extensor tendon [56]. At the midpoint of the proximal phalanx, the compact bone layer was of remarkable thickness and formed a ring of low intensity surrounding a hyperintense fatty marrow [56].

Our study reported that on the plantar aspect of the metatarsal bones, the interosseous muscle (IOM) appeared conspicuous and appeared as a flattened cord. The superficial digital flexor tendon (SDFT) and deep digital flexor tendon (DDFT) were also readily identifiable on all planes of CT images and sectional anatomy. Moreover, the SDFT and DDFT were smoothly marginated along the plantar aspect of the IOM and the fused metatarsal bones. Each tendon was surrounded by a small rim representing its tendon sheath and bifurcated into medial and lateral limbs proximal to the fetlock joint. Each limb of the SDFT received a connecting band from the IOM, forming a sleeve around the corresponding band of the DDFT proximal to the metatarsophalangeal joint. Also, distal to the proximal sesamoid bones and prior to its insertion in the base of the middle phalanx, SDFT gained a position deeper than that of DDFT. Our study reported that each of the medial and lateral collateral tarsal ligaments detached the long part and short part (MCTLL, MCTLs, LCTLL, and LCTLs, respectively). Moreover, the digital ligaments, including collateral, proximal interdigital, suspensory, plantar, interdigital inter-sesamoidean, collateral sesamoidean, and interdigital phalango-sesamoidean, were well outlined on coronal and transverse CT images, while the plantar annular ligament was best delineated on a sagittal CT image. However, the short and cruciate sesamoidean ligaments could not be outlined on CT images.

The 3D render volume reconstruction of the pes region of the Zebu cattle allowed us to see the pes' bones from all angles and slightly and provided a chance to explore the articular surfaces of the different parts of the bones of the pes region. Radiologists can identify and highlight personal skeletal and soft-tissue characteristics of various anatomy using 3D reformatted images, which improve temporal bone anatomy explanation and have the ability to evaluate related diseases [61, 62]. With the help of 3D imaging technology, anatomical models can be produced on a 3D printer to aid in learning, improved comprehension, research, and diagnosis [12, 13, 63, 64].

Finally, Zebu cattle are more prone to joint lameness than other Egyptian cattle species, even though they have adapted exceptionally well to the country's environment. This susceptibility stems from a variety of factors and conditions. Correct arthrocentesis of affected joints requires a thorough understanding of the PES region, the cattle's joints, and the topographical and anatomical characteristics of the Zebu breed, as described in the camel [12, 13]. Our data is useful for surgeons and students to understand that there is one factor contributing to the joint lameness in Zebu cattle: their heavier body weight in the weal PES region compared to other Egyptian cattle species. This puts more stress on their joints, making them more susceptible to lameness. Additionally, the Zebu breed's genetic predisposition towards certain joint disorders can also contribute to their increased vulnerability to joint lameness.

In our future research, we will focus on the study of both hard and soft tissues and the PES musculature and vasculature to provide a more comprehensive idea about the PES region's function and potential vulnerabilities. Additionally, we will explore the interaction between the PES region and adjacent anatomical structures to better understand its role in overall biomechanics and potential implications for surgical interventions. Furthermore, our research will investigate potential diagnostic techniques and therapeutic approaches that can target specific vulnerabilities in the PES region for improved patient outcomes.

## Conclusion

The study analyzed the pes of cattle from Zebu bulls using various methods, including cross-sectional anatomy, CT, 3D render volume reconstruction CT, and radiographics, to provide detailed images of the PES region. The study aimed to provide anatomical guidance for surgeons and students in PES regions. The radiographic and CT images could be used as a reference for interpreting clinical diseases in the PES. A radiographic examination revealed two large fused metatarsal bones that were completely fused except for their distal extremities. The bony septum divided the medullary cavity between the two fused large metatarsal bones in the proximal distal half and disappeared in the middle part. Our study serves as a foundation for diagnosing PES region disorders using CT imaging and 3D reconstructions.

## Data Availability

The datasets used and/or analyzed during the current study are available from the corresponding author on reasonable request. Research data are not shared.
